# Overexpression of Krüppel-Like Factor 4 Suppresses Migration and Invasion of Non-Small Cell Lung Cancer Through c-Jun-NH2-Terminal Kinase/Epithelial-Mesenchymal Transition Signaling Pathway

**DOI:** 10.3389/fphar.2019.01512

**Published:** 2020-01-08

**Authors:** Yanping Wu, Lianjun Lin, Xiang Wang, Yong Li, Zhonghui Liu, Wei Ye, Weiming Huang, Gang Lin, Haibo Liu, Jixin Zhang, Ting Li, Beilei Zhao, Liping Lv, Jian Li, Nanping Wang, Xinmin Liu

**Affiliations:** ^1^Department of Geriatrics, Peking University First Hospital, Beijing, China; ^2^Department of Interventional Pulmonary Disease, Anhui Chest Hospital, Hefei, China; ^3^Department of Thoracic Surgery, Peking University First Hospital, Beijing, China; ^4^Department of Pathology, Peking University First Hospital, Beijing, China; ^5^Key Laboratory of Molecular Cardiovascular Science of Ministry of Education, Peking University Health Science Center, Beijing, China

**Keywords:** invasion, Krüppel-like factor 4, metastasis, migration, non-small cell lung cancer

## Abstract

Krüppel-like factor 4 (KLF4) is a transcription factor and plays a vital role in cancer initiation and development. However, the role of Krüppel-like factor 4 in the metastasis of non-small cell lung cancer (NSCLC) is not clear. Here, we demonstrated that the expression of Krüppel-like factor 4 was significantly decreased in human non-small cell lung cancer tissues compared with that in normal tissues using Western blot. We performed immunohistochemical staining and observed the decreased expression of Krüppel-like factor 4 in human lung cancer tissues, and metastatic tumor tissues located in the trachea and main bronchus. We also found that the E-cadherin expression was decreased, while vimentin expression was increased in human NSCLC tissues and metastatic tumor tissues located in the trachea and main bronchus. Additionally, enforced expression of Krüppel-like factor 4 in mouse lungs significantly inhibited the metastasis of circulating Lewis lung carcinoma cells to the lungs by attenuating mesenchymal-epithelial transition (MET). Furthermore, cell scratch assays and Matrigel invasion assays revealed that overexpression of Krüppel-like factor 4 inhibited the migration and invasion of non-small cell lung cancer cell lines A549, H1299, H226, and H1650 cells. Moreover, overexpression of Krüppel-like factor 4 attenuated TGF-β1-induced epithelial-mesenchymal transition (EMT) in A549, and inhibited the phosphorylation of c-Jun-NH2-terminal kinase (JNK), an important pathway in metastasis in non-small cell lung cancer. Our *in vivo* and *in vitro* findings illustrate that Krüppel-like factor 4 inhibited metastasis and migration of non-small cell lung cancer, and indicate that Krüppel-like factor 4 could be a potential therapeutic target for the treatment of non-small cell lung cancer.

## Introduction

Lung cancer is the leading cause of cancer death in men and the second leading cause of cancer in women worldwide (Torre et al., 2015). The incidence and mortality rate of lung cancer have been increasing in China along with economic development, air pollution, tobacco, and occupational exposures (Chen et al., 2016). Despite advances in the diagnosis and treatment of lung cancer, the prognosis of lung cancer is still poor, and the 5-year survival rate remains low due to the lack of early diagnoses. Non-small cell lung cancer (NSCLC) accounts for more than 85% of all lung cancer cases. However, approximately 70% of patients with lung cancer present with locally advanced or metastatic disease at the time of diagnosis (Molina et al., 2008). Further exploring the metastatic mechanism and finding a potential therapeutic target for NSCLC are of vital and urgent significance.

Krüppel-like factor 4 (KLF4) is a member of the KLF family, with an evolutionarily conserved mammalian zinc finger structure. Since 2005, seventeen members of the KLF family have been discovered (Suske et al., 2005), which are involved in many physiological processes, such as embryonic development (Zhang et al., 2010), organ formation, stemness (Katz et al., 2002; Jaubert et al., 2003; Takahashi and Yamanaka, 2006; Kim et al., 2008), and aging (Wong et al., 2010; Panatta et al., 2018). They are also involved in pathophysiological processes, such as tumorigenesis, inflammation and radiation injury (Li et al., 2012; Zahlten et al., 2013; Wei et al., 2016; Kuruvilla et al., 2016). KLF4 also plays a vital role in pulmonary hypertension (Shatat et al., 2014; Ban et al., 2019) and pulmonary fibrosis. Our previous work showed that KLF4 attenuated lung fibrosis *via* an inhibition of epithelial-mesenchymal transition (EMT) (Lin et al., 2017), which also played an important role in cancer metastasis. Li et al. found that KLF4 inhibited invasion and metastasis *via* suppressing MMP2 promoter activity (Li et al., 2017). Zhou et al. discovered that ectopic expression of KLF4 downregulated SPARC gene expression to inhibit cell invasion (Zhou et al., 2010). Vaira V. et al. revealed that Numbl-Klf4 signaling plays an important role in metastatic progression (Vaira et al., 2013). Previous studies found that KLF4 may function as a tumor suppressor gene in lung cancer; however, its role in the metastasis and invasion of lung cancer remains unclear and needs further exploration. APTO-253 is an inducer of KLF4 in human colon, NSCLC, breast, prostate, and acute myelogenous leukemia cell lines. It was used in phase I clinical trial, in patients with advanced or metastatic solid tumors. Results showed that APTO-253 was well tolerated and could stable disease (Cercek et al., 2015). Then our study aimed to further investigate how upregulation of KLF4 affects NSCLC metastasis.

## Materials and Methods

### Sample Collection

Human lung tissues were obtained from NSCLC patients undergoing surgery in the Department of Thoracic Surgery at the Peking University First Hospital. Twenty-one pairs of NSCLC tissues and matched adjacent nontumor lung tissues were collected to detect the protein expression of KLF4. Forty formalin-fixed paraffin-embedded human NSCLC samples were collected from the Department of Pathology at the Peking University First Hospital. Twelve paracancerous normal lung tissue samples were collected for use as control specimens. All tumor and normal tissues were obtained from the surgical specimens of patients with NSCLC. Twelve airway tumor tissues were collected from the Interventional Pulmonary Disease Department at the Anhui Thoracic Hospital. These tissues were fixed in formalin, and immunohistochemistry was applied to detect KLF4 expression. This study was approved by the Clinical Research Ethics Committee of the Peking University First Hospital, Beijing, China. Ethical review number is No. (2019) SCI (91).The informed consents obtained from participants were both informed and written. The participants must be above the age of 18, diagnosed with non-small cell lung cancer with complete clinical data, and signed the informed consent.

### Adenoviral Vectors and Infections

The KLF4 adenovirus was constructed as previously described (Wang et al., 2002; Wang et al., 2012). The expression of the inserted KLF4 was driven by a 7 × tet operon/minimal cytomegalovirus promoter, which was further under the control of a tetracycline-controlled transactivator (tTA). The adenoviruses were purified by cesium chloride methods. For adenovirus-mediated gene transfer, confluent cell lines were exposed to adenoviral vectors with tetracycline transactivator adenovirus (Ad-tTA) to induce tetracycline-controllable expression. Cells were co-infected with AdKLF4 and AdtTA (20 MOI) and incubated for 6 h with or without tetracycline (0.1 μg/ml).

### *In Vivo* Lung Metastatic Tumor Mouse Model

Specific pathogen-free seven-week-old female C57BL/6J mice were housed under barrier conditions. Furthermore, we constructed an adeno-associated viral vector, AAV5, with a stable and highly efficient KLF4 expression and an enforced green fluorescent protein (GFP) expression. Mice were divided into two groups and injected with AAV5-KLF4 (1.75 × 10^13^ v.g/ml) or CON-AAV5 (1.80 × 10^13^ v.g/ml) through the trachea using a microsprayer (1.5 × 10^11^ v.g/mouse). Two weeks later, mice received tail venous injections of 1 × 10^6^ Lewis lung carcinoma (LLC) cells that had firefly luciferase as a reporter enzyme. We utilized *in vivo* imaging systems (IVIS) in conjunction with D-luciferin to progressively track the invasion of the LLC cells. All images were acquired 4–6 min after the intraperitoneal injection of D-luciferin potassium salt solution (150 mg/kg, Cat. No. 7903, Biovision, San Francisco, USA). A mouse was anesthetized with an intraperitoneal injection of 2,2,2-tribromoethanol (Product Number T48402, Sigma-Aldrich, St. Louis, USA) and placed in a dark chamber during image acquisition. Bioluminescence imaging was immediately performed after injections of the LLC cells, and on days 7, 14, 18, and 21. Mice were euthanized three weeks later, and all animal protocols were reviewed and approved by the Animal Research Committee of the Peking University First Hospital. At the end of the experiment, the lungs were collected and fixed in 10% formalin solution for hematoxylin and eosin staining and to directly view GFP expression under a fluorescence microscope.

### Cells and Cell Culture

The NSCLC cell line A549 was purchased from the American Type Culture Collection (Manassas, VA, USA), and the NCI-H1299 cell line was obtained from the Peking Union Medical College (Beijing, China). The NCI-H226 human squamous carcinoma cell line and NCI-H1650 human cancer cell line were purchased from the Cell Bank of Shanghai Institute of Cell Biology. Lewis lung carcinoma (LLC) cells with a luciferase reporter gene were constructed and purchased from Shanghai Genechem Company. The A549 cells were cultured in Dulbecco’s modified Eagle’s medium (DMEM), and the other cells were cultured in Roswell Park Memorial Institute (RPMI) 1640 medium supplemented with 10% fetal bovine serum (FBS) (Gibco, MA, USA) at 37°C in 5% CO_2_.

### Immunohistochemistry and Immunofluorescence

Immunohistochemical staining was performed using Biotin-Streptavidin horseradish peroxidase (HRP) Detection Systems (SP-9000, ZSGB-BIO, Beijing, China). The tissue sections were incubated with 3% H_2_O_2_ deionized water for 10 min and blocked with goat serum (SP-9000, Reagent A) at room temperature. Then, the tissue sections were incubated with rabbit polyclonal anti-KLF4 (1:2,000 dilution, Cat. No. ab215036; San Francisco, Abcam), rabbit polyclonal anti-E-cadherin (1:400 dilution, Cat. No. 3195T; Danvers, CST), rabbit polyclonal anti-vimentin (1:3,000 dilution, Cat. No. 10366-1-AP; Rosemont, Proteintech) primary antibodies overnight at 4°C.

Immunofluorescence staining was performed to detect KLF4, E-cadherin, and vimentin expression. The tissue sections were blocked with 5% bovine serum albumin (BSA) and incubated with rabbit monoclonal anti-KLF4 (1:500 dilution, Cat. No. ab214666; San Francisco, Abcam), rabbit polyclonal anti-E-cadherin (1:200 dilution, Cat. No. 3195T; Danvers, CST), and rabbit monoclonal anti-vimentin (1:400 dilution, Cat. No. ab92547; San Francisco, Abcam) primary antibody overnight at 4°C separately. The tissue sections were incubated with GoatAnti-Rabbit IgG H&L(HRP) (1:300 dilution, Cat. No. ab6721; San Francisco, Abcam) second antibody at room temperature for 1 h.

### Evaluation of Immunohistochemical Staining

A quantitative score was estimated by adding the score of the staining area and that of staining intensity for each case to assess the expression levels of the protein. The quantitative score was estimated by calculating the percentage of immunopositive cells as follows: 0, no staining of cells in any microscopic fields; 1+, < 30% of cells stained positive; 2+, 30-60% stained positive; and 3+, > 60% stained positive. The intensity was scored by evaluating the average staining intensity of the positive cells as follows: 0, no staining; 1+, mild staining; 2+, moderate staining; and 3+, intense staining. Evaluation of the stained sections was performed by two pathologists blinded to the clinical information. Each section was scanned at 20× and 40× magnification by light microscopy.

### *In Vitro* Cell Scratch Assay

For the cell motility assay, the transfected cells were seeded in 6-well plates and cultured when almost confluent. A crossed wound was created by scratching the confluent monolayer of the cells using a 10 µl sterile pipette tip. The cells were washed three times with phosphate-buffered saline (PBS) and incubated with DMEM or RPMI 1640 supplemented with 0.2% FBS. Images were taken at 0 h and 24 h after wounding under inversion fluorescence microscope (Olympus, Tokyo, Japan). We used ImageJ software (National Institutes of Health, USA) to assess the area of the wound at 0 h and 24 h, and the wound closing area was calculated by the wound area at 24 h minus the wound area at 0 h.

### *In Vitro* Cell Matrigel Invasion Assay

Transwell chamber inserts (8 μm pore size, Corning, New York, USA) coated with 200 μg/ml of mixed Matrigel (Cat. No. 354234, Corning, New York, USA) were used for the cell invasion assay. The transfected cells were implanted at 5 × 10^4^ cells/well in 200 μl of serum-free medium. A total of 750 μl of complete medium supplemented with 10% FBS was added to the bottom of the inserts, allowing the cells to invade for 24–48 h. After incubation, the cells on the upper surface of the membrane were removed, while those on the lower filter surfaces were fixed and stained with crystal violet. The number of invaded cells was counted under a microscope.

### Western Blot Analysis

Total protein was extracted from human tissues and cell lines using protein extraction buffer containing a 1% protease inhibitor cocktail (Targetmol, Shanghai, China) and a phosphatase inhibitor (Cat. No. 4906845001, Roche, Basel, Switzerland). The lysate was centrifuged at 4°C at 14,000 rpm for 15 min, and proteins were separated by 10% sodium dodecyl sulfate polyacrylamide gel electrophoresis (SDS-PAGE) and transferred to polyvinylidene fluoride membranes (PVDF) (Millipore, Massachusetts, USA). After being blocked with 5% skim milk for 1 h at room temperature, the membranes were incubated at 4°C overnight with primary KLF4 (ab215036, Abcam, San Francisco, USA), p-JNK (sc-293136, Santa, California, USA), and JNK (sc-7345, Santa, California, USA) antibodies and glyceraldehyde-3-phosphate dehydrogenase (GAPDH) (TA-08, ZSGB, Beijing, China) antibodies. After washing with Tris-buffered saline with 1% Tween-20 (TBST), the membranes were incubated with HRP-conjugated secondary antibodies (ZSGB-Bio, Beijing, China) for 1 h at room temperature. The blots were visualized using enhanced chemiluminescence reagents (ECL) detection (Amersham Biosciences Fairfield, CT, USA).

### Statistical Analysis

All statistical analyses were carried out by SPSS software, version 24.0. Differences among sample groups were analyzed using independent *t*-tests or nonparametric tests. For the migration and invasion assays and the immunohistochemical evaluation, the independent *t*-test method was used. For the Western blotting examination of KLF4, E-cadheirn, and viemntin expression, Wilcoxon signed-rank test was used. The nonparametric test was used to compare the total luminescence of (region of interests [ROIs]; lungs) between the control and AAV5 groups. A p < 0.05 was considered statistically significant.

## Results

### The Expression of Krüppel-Like Factor 4 Was Decreased in Human Non-Small Cell Lung Cancer Tissues

**Figure 1:**
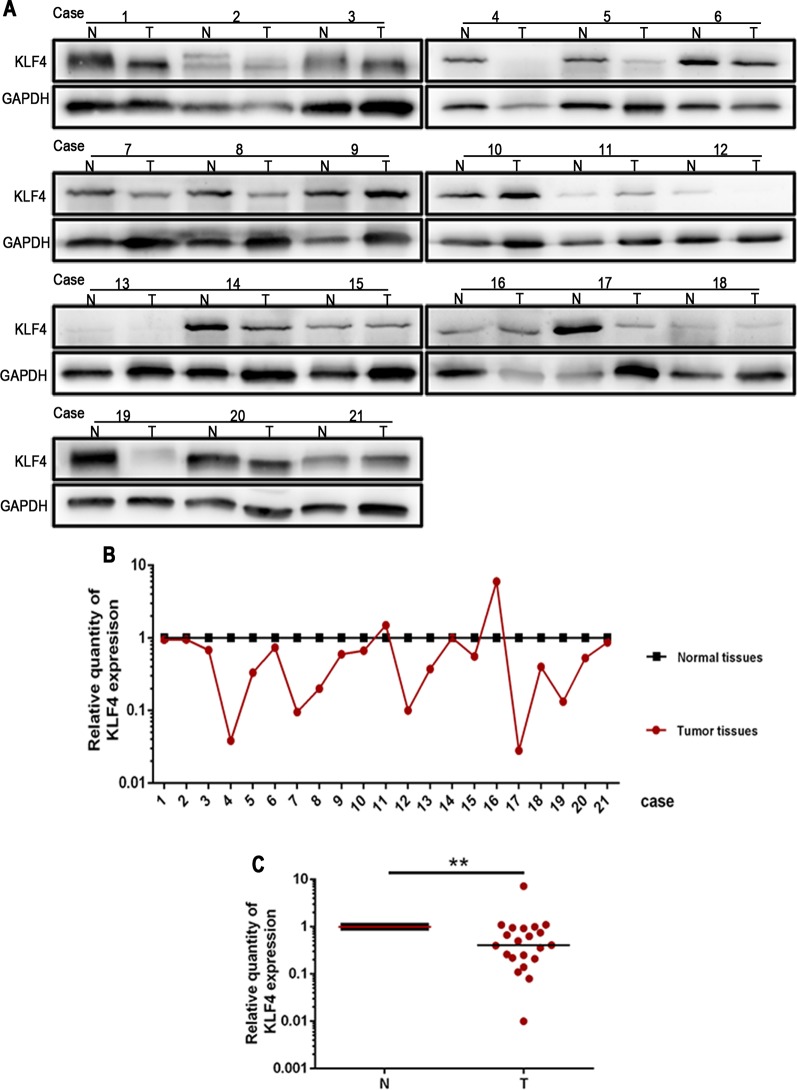
Krüppel-like factor 4 (KLF4) expression was decreased in human non-small cell lung cancer (NSCLC) tissues. **(A)** Western blot of KLF4 in human NSCLC tissues and adjacent normal tissues from the individual patient. GAPDH was used as a loading control. **(B)** The quantitation of KLF4 expression in each sample was normalized with that of GAPDH by determining the KLF4/GAPDH ratio. N; Normal. T; Tumor. **(C)** Quantitative analysis demonstrating a significant decrease of KLF4 expression in the tumor tissues compared with adjacent normal tissues. Data presented are medians. **p < 0.05.

The expression of KLF4 was examined using Western blotting in 21 paired human NSCLC tissues and adjacent normal tissues. The results showed that the expression of KLF4 was downregulated in NSCLC tissues compared with adjacent normal tissues (**Figure 1**).

**Figure 2:**
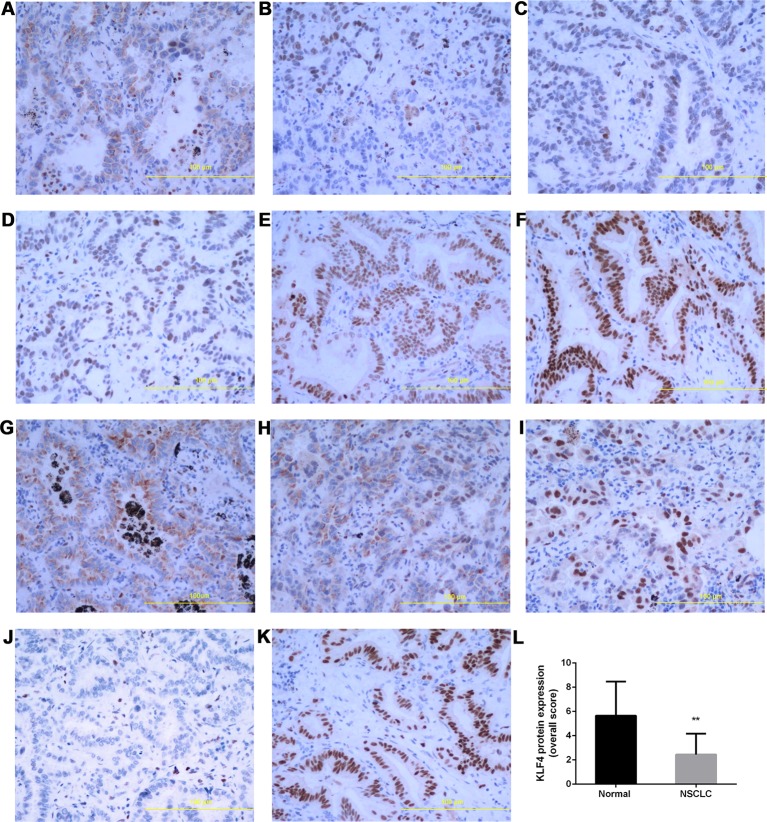
Krüppel-like factor 4 (KLF4) expression was decreased in human non-small-cell lung cancer (NSCLC) tissues. Immunohistochemistry was performed to evaluate the expression of KLF4. Tissue sections with **(A)** <30% of cells stained positive in the microscopic field, **(B)** 30-60% stained positive, and **(C)** >60% stained positive. Tissue sections with **(D)** mild staining, **(E)** moderate staining, and **(F)** intense staining. **(G–I)** Subcellular localization of KLF4.Tissue sections with **(G)** positive staining in the cytoplasm, **(H)** positive staining in the nucleus and cytoplasm, **(I)** positive staining in the nucleus. **(J)** No positive staining in the nucleus and cytoplasm. **(K)** KLF4 staining in adjacent normal tissues. **(L)** Quantitative analysis demonstrating a signiﬁcant decrease of KLF4 expression in the tumor tissues compared with adjacent normal tissues. Data presented are means ± SD. **p < 0.05.

The expression of KLF4 in lung cancer tissues was further demonstrated using immunohistochemistry. Tissue sections from 40 human NSCLC and 12 adjacent normal tissues were used to observe the expression of KLF4. The positive pattern of KLF4 staining was a yellow or brownish-yellow stain, mainly in the nuclei. The percentage of KLF4 positive staining is presented in **Figures 2A**–**C**; the intensity of KLF4 staining is presented in **Figures 2D**–**F**. No positive staining was shown in **Figure 2J**. **Figure 2K** was the representative image of KLF4 staining in adjacent normal tissues. Liu et al. found that subcellular localization of KLF4 is associated with the prognosis of NSCLC patients (Liu et al., 2018). Among the tissue sections we collected, only a few tissues were KLF4 positive staining in both cytoplasma and nuclei. (**Figure 2G–I**; the subcellular localization of KLF4.) We assessed the KLF4 positive staining in nuclei using a quantitative score evaluating system and the statistics analysis of KLF4 expression in cancer tissues was lower than that in normal tissues, as shown in **Figure 2L**. All of the data showed that the expression of KLF4 was downregulated in the NSCLC tissues compared with the adjacent normal tissues.

**Figure 3:**
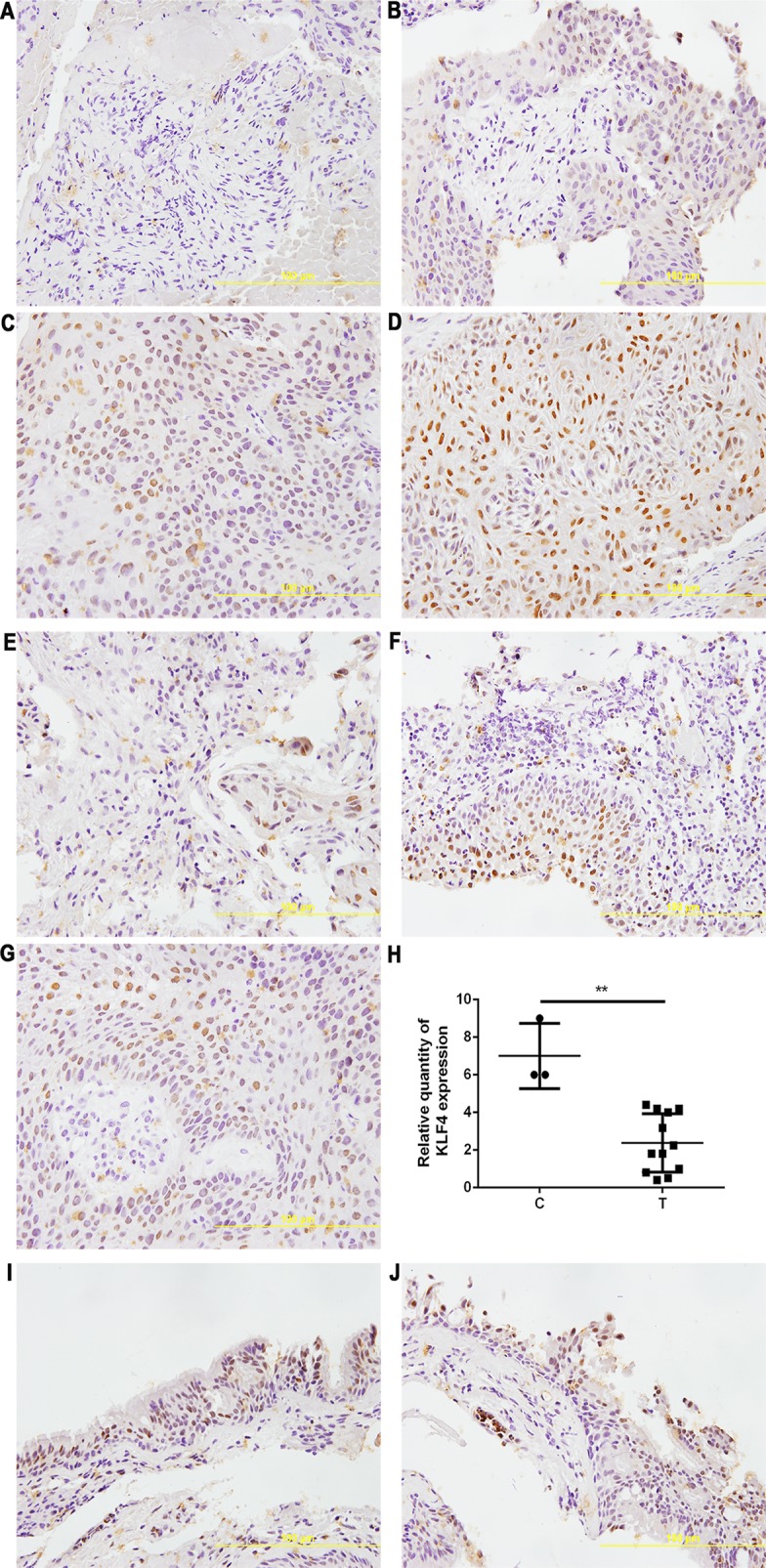
Krüppel-like factor 4 (KLF4) expression was decreased in human airway metastatic tumor tissues. Immunohistochemistry was performed to evaluate the expression of KLF4. Tissue sections with **(A)** no staining, **(B)** mild staining, **(C)** moderate staining, and **(D)** intense staining. Tissue sections with **(E)** < 30% of cells stained positive in the microscopic field, **(F)** 30-60% stained positive, and **(G)** > 60% stained positive. **(H)** Quantitative analysis demonstrating a significant decrease of KLF4 expression in the tumor tissues compared with adjacent bronchial epithelial cells. Data presented are medians. **p < 0.05. C, control; T, tumor tissues. **(I,J)** KLF4 staining in adjacent bronchial epithelial tissues as control.

We also detected the expression of KLF4 in metastatic tumor tissues located in the trachea and main bronchus from 12 cases with NSCLC by immunohistochemistry. As shown in **Figure 3**, the intensity (**Figures 3A**–**D**) and percentage (**Figures 3E**–**G**) of KLF4 positive staining was varied in the metastatic tumor tissues located in the trachea and main bronchus. **Figure 3H** showed that KLF4 expression was decreased in tumor tissues compared with adjacent bronchial epithelial tissues. The positive staining of KLF4 in adjacent bronchial epithelial tissues was shown in **Figures 3I**, **J**. Hence, we assumed that KLF4 may affect the tumor metastasis and distant invasion.

### E-Cadherin Expression Was Decreased and Vimentin Expression Was Increased in Human Non-Small Cell Lung Cancer Tissues

**Figure 4:**
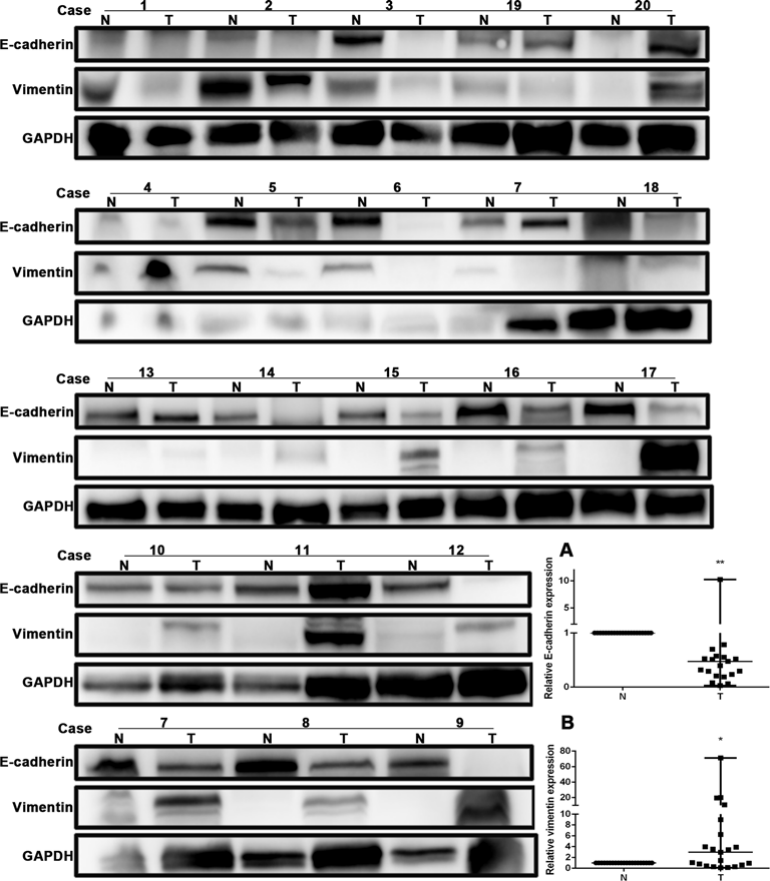
E-cadherin expression was decreased, while vimentin expression was increased in human non-small-cell lung cancer (NSCLC) tissues. Western blot was performed in human NSCLC tissues and adjacent normal tissues from the individual patient. GAPDH was used as a loading control. The quantitation of E-cadherin and vimentin expression in each sample was normalized with that of GAPDH by determining the E-cadherin/GAPDH and vimentin/GAPDH ratio. N, Normal. T, Tumor. **(A)** Quantitative analysis demonstrating a significant decrease of E-cadherin expression in the tumor tissues compared with adjacent normal tissues. Data presented are medians. **p < 0.05. **(B)** Quantitative analysis demonstrating a significant increase of vimentin expression in the tumor tissues compared with adjacent normal tissues. Data presented are medians. *p < 0.05.

**Figure 5:**
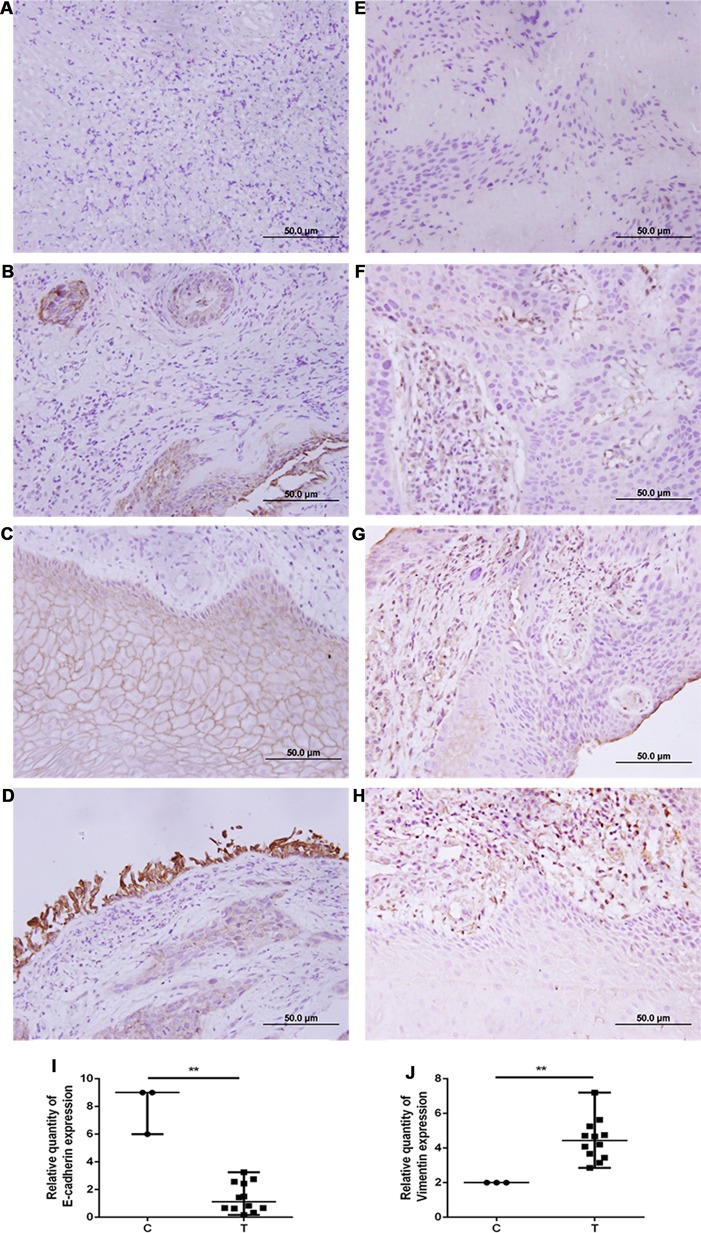
E-cadherin expression was decreased, while vimentin expression was increased in human airway metastatic tumor tissues. Immunohistochemistry was performed to evaluate the expression of E-cadherin **(A**–**D)** and vimentin **(E**–**H)**. Tissue sections with **(A**, **E)** no staining, **(B**, **F)** mild staining, (**C**, in the upper middle of the image) **(G)** moderate staining, and **(D**, **H)** intense staining. **(I**, **J)** Quantitative analysis demonstrating a significant decrease of E-cadherin expression and a significant increase of vimentin expression in the tumor tissues compared with adjacent bronchial epithelial cells. Data presented are medians. **p < 0.05. C, control; T, tumor tissues.

The expression of E-cadherin and vimentin was detected in 21 paired human NSCLC tissues and adjacent normal tissues by Western blotting. The results showed that the expression of E-cadherin was decreased in NSCLC tissues compared with adjacent normal tissues, as shown in **Figure 4A**. The expression of vimentin was increased in NSCLC tissues compared with adjacent normal tissues, as shown in **Figure 4B**. The expression of E-cadherin and vimentin was also detected in metastatic tumor tissues located in the trachea and main bronchus from 12 cases with NSCLC by immunohistochemistry. The intensity of E-cadherin (**Figures 5A–D**) and vimentin (**Figures 5E–H**) positive staining was shown in **Figure 5**. **Figures 5I**, **J** showed that E-cadherin expression was decreased, while vimentin expression was increased in tumor tissues compared with adjacent bronchial epithelial tissues.

### Overexpression of Krüppel-Like Factor 4 Suppressed Tumor Metastasis by Attenuating MET *In Vivo*

**Figure 6:**
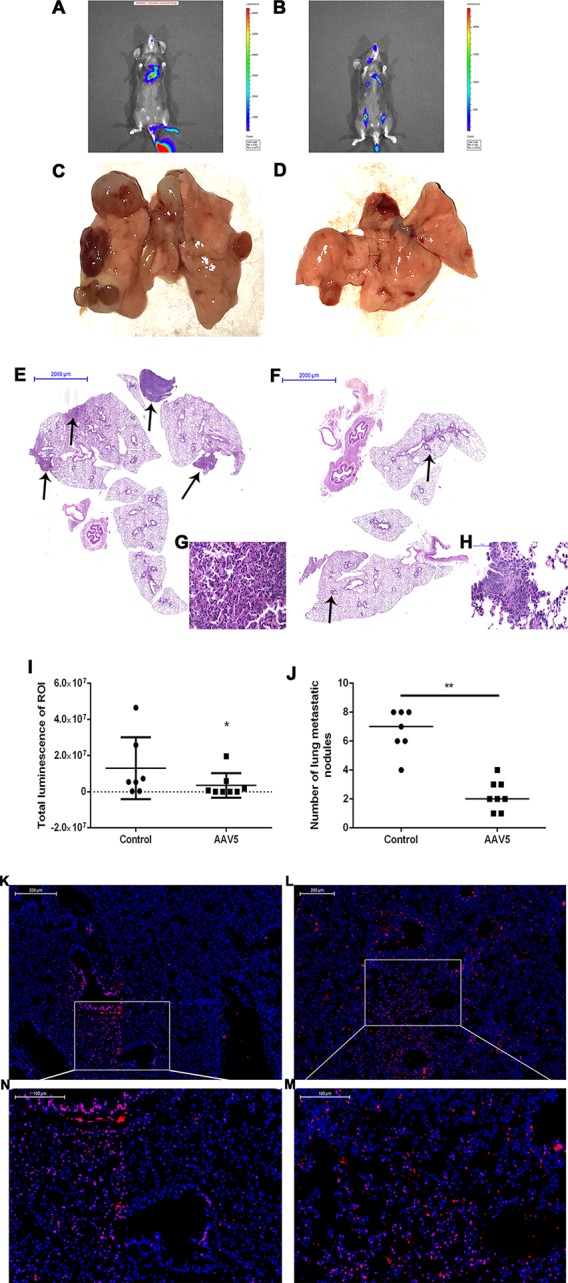
Enforced expression of Krüppel-like factor 4 (KLF4) in the lung suppressed tumor metastasis and invasion *in vivo*. **(A, B)** Bioluminescence was analyzed in mice after tail vein injection of Lewis lung cancer (LLC) cells (n = 8 per group). Whole body bioluminescent imaging of animals was acquired on day 21 after tumor cell injection. **(I)** The total luminescence of lung between the control and AAV5 groups was compared by nonparametric tests (*p < 0.05). **(C, D)** Images of the lungs were taken after mice were sacrificed. **(J)** The number of metastatic nodules on the surface of the lungs was compared by nonparametric tests between the control adeno-associated viral vector (AAV5) and AAV5 expressing KLF4 groups (**p < 0.05). **(E–H)** Hematoxylin and eosin staining of mouse lungs at different magnifications. The arrow shows the location of the metastatic tumor. **(G,H)** The typical magnified image of a mouse metastatic tumor. **(K)** Immunofluorescence staining for KLF4 in paraffin sections from mouse lungs infected by control AAV5. **(L)** Immunofluorescence staining for KLF4 in paraffin sections from mouse lungs infected by AAV5 expressing KLF4. **(M,N)** The magnified image of the indicated area in **(K,L)** separately.

**Figure 7:**
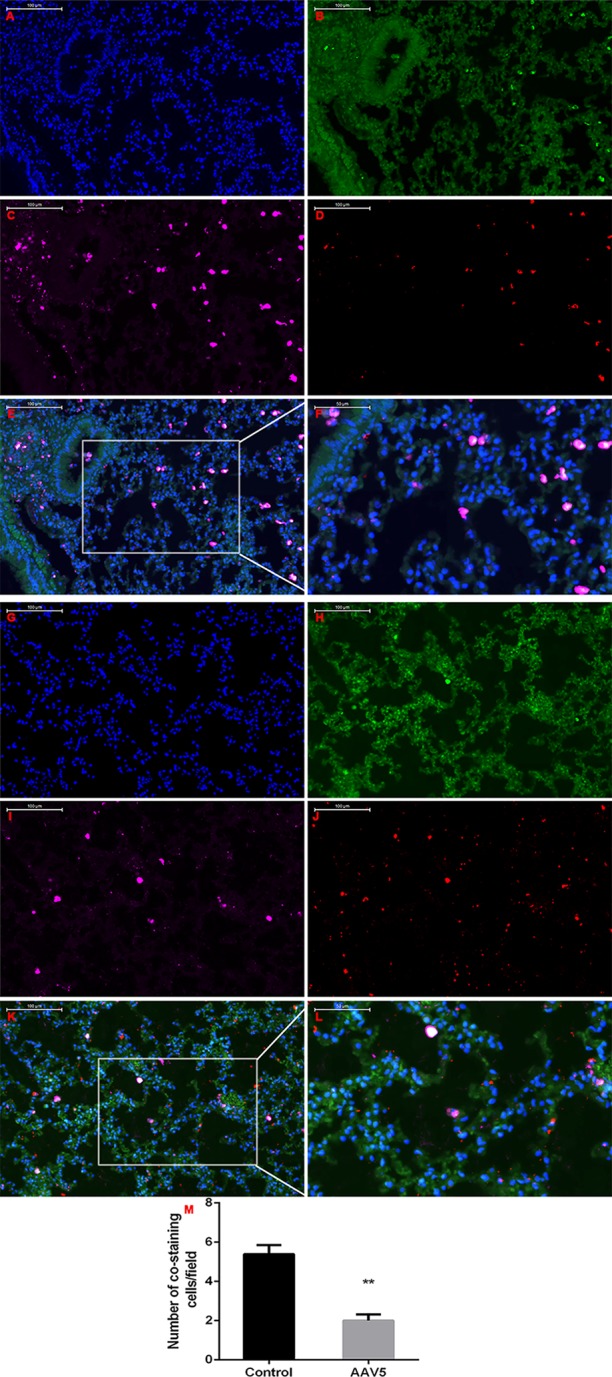
Enforced expression of Krüppel-like factor 4 (KLF4) in the lung attenuated MET *in vivo*. Triple-labeled immunofluorescence staining were performed to examine the expression of KLF4 (red), E-cadherin (green), and vimentin (pink) in mouse lungs. **(A**–**F)** Typical images of control AAV5 vector group, and **(G**–**L)** typical images of AAV5 expressing KLF4 group. **(M)** The number of triple-labelled positive cells was calculated in each group. The values were shown as mean ± SD. **P < 0.05 by *t*-test.

To determine the role of KLF4 on tumor metastasis *in vivo*, enforced KLF4 expression was conducted in the lungs of mice *via* an adeno-associated viral vector type 5 (AAV5), which was labeled with GFP (green fluorescent protein). A mouse model of lung cancer metastasis was used through intravenous injection of LLC cells with a luciferase reporter gene. The metastasis of lung cancer cells was confirmed by IVIS (*in vivo* imaging systems) and pulmonary pathology. The bioluminescent intensity of mice on day 21 was reduced in the AAV5-KLF4 group than in the AAV5-control group (**Figures 6A**, **B**, **I**; the average rank of the total luminescence of the lungs was 10.43 *vs.* 5.88.). **Figures 6C**, **D**, **J** show the number of metastatic tumors on the surface of mouse lungs in the AAV5-KLF4 group was less than that in the AAV5-control group. The pathology of the lungs was analyzed after the mice were sacrificed. The hematoxylin and eosin staining of lung tissue sections showed the number of metastatic tumors in the AAV5-KLF4 group and in the AAV5-control group (**Figures 6E**–**H**). Images in **Figures 6K**, **L** show the KLF4 expression in lung tissues by immunofluorescence staining, which confirmed the successful infection of AAV5. **Figure 4N**, **M** were magnified images of the indicated area of **Figure 6K**, **L**. To assess the presence of MET in lung metastasis mouse model, expression levels of KLF4, E-cadherin and vimentin were analyzed in sections of lung tissues from mice in control-AAV5 group and AAV5-KLF4 group with triple-labeled immunofluorescence staining method. Triple stained cells were detected in mouse lungs. Less triple positive cells were observed in AAV5-KLF4 group (**Figure 7K**) per field compared with control-AAV5 (**Figure 7E**) group. Overexpression of KLF4 significantly decreased the number of cells undergoing MET in lung metastasis mouse model (**Figure 7M**). These results showed that overexpression of KLF4 in the lungs inhibited the metastasis of circulating LLC cells to the lungs by attenuating MET *in vivo*.

### Overexpression of Krüppel-Like Factor 4 Attenuated the Migration and Invasion of Cancer Cells *In Vitro*

**Figure 8:**
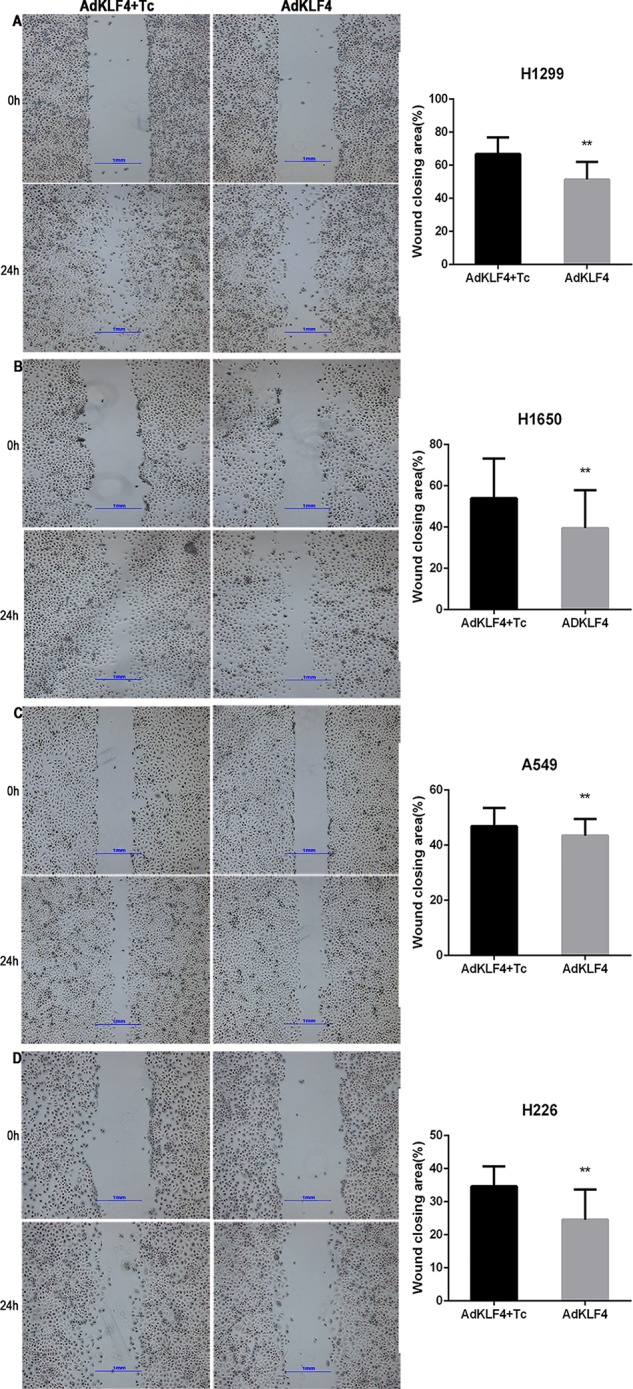
Ectopic expression of Krüppel-like factor 4 (KLF4) suppressed lung cancer cell migration. **(A**–**D)**. **(A)** H1299, **(B)** H1650, **(C)** A549, and **(D)** H226 lung cancer cells were subjected to a wound-healing assay. Representative areas of migrated cells and wound closing area (%) are shown (mean ± SEM). **p < 0.05. The experiment was repeated at least three times. Representative photographs were taken at 4× magnification. AdKLF4, Lung cancer cells were transfected with adenovirus expressing KLF4. AdKLF4+Tc, Lung cancer cells were transfected with adenovirus expressing KLF4 and cultured with tetracycline.

**Figure 9:**
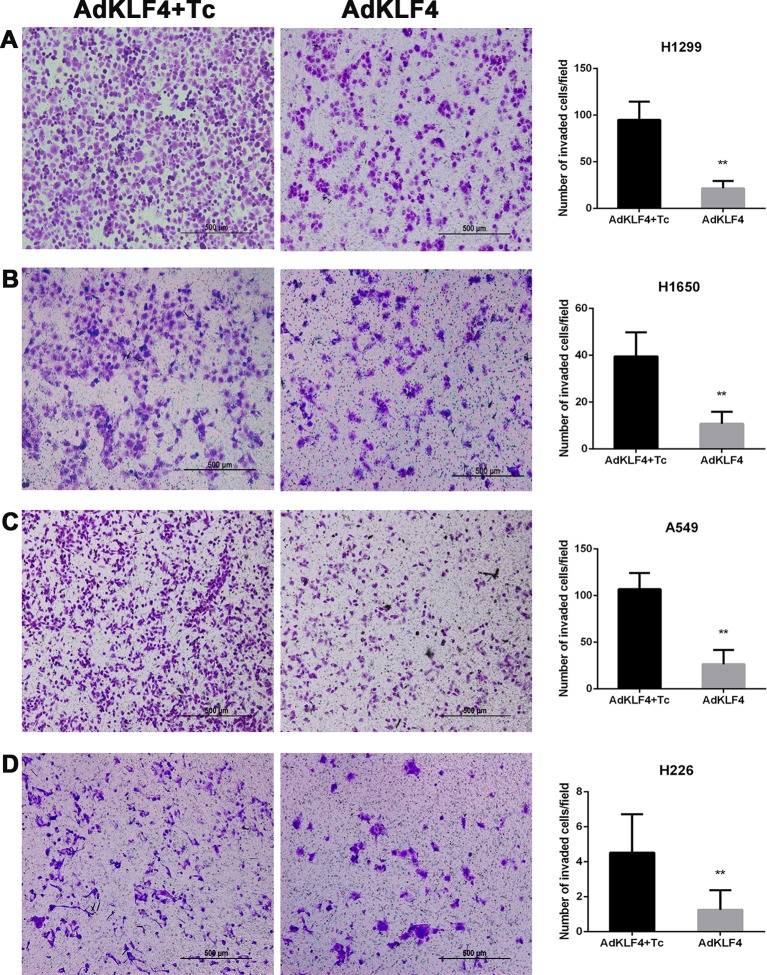
Ectopic expression of Krüppel-like factor 4 (KLF4) suppressed lung cancer cell invasion **(A**–**D)**. **(A)** H1299, **(B)** H1650, **(C)** A549, and **(D)** H226 lung cancer cells were subjected to the transwell migration assay. Representative areas of invaded cells and the number of invaded cells/field are shown (mean ± SEM). **p < 0.05. The experiment was repeated at least three times. AdKLF4: Lung cancer cells were transfected with adenovirus expressing KLF4. AdKLF4+Tc: Lung cancer cells were transfected with adenovirus expressing KLF4 and cultured with tetracycline.

To determine the effect of KLF4 on the migration and invasion of lung cancer cells, A549, H1299, H226, and H1650 cells were exposed to adenoviral vectors with tetracycline transactivator adenovirus (Ad-tTA) to induce tetracycline-controlled expression. Infected cells were scratched, washed and cultured at 37°C for additional 24 h with or without tetracycline. As shown in **Figure 8**, the wound scratch assay indicated that the overexpression of KLF4 attenuated the migration of A549, H1299, H226, and H1650 cells (46.85 ± 0.7672 *vs.* 43.46 ± 0.7094, 66.76 ± 1.795 *vs.* 51.34 ± 1.860, 34.64 ± 1.028 *vs.* 24.55 ± 1.564, and 53.94 ± 2.579 *vs.* 39.51 ± 2.451, respectively). Second, NSCLC cells were analyzed in a Matrigel invasion assay. A total of 2 × 10^4^ infected cells were seeded in an invasion chamber for additional 24 to 48 h. Invaded cells on the lower surface of the chamber were stained and counted under a 40× light microscope. **Figure 9** shows that the overexpression of KLF4 delayed the invasion of A549, H1299, H226 and H1650 cell lines (106.9 ± 2.752 *vs.* 26.40 ± 2.423, 94.92 ± 2.534 *vs.* 21.57 ± 1.030, 4.526 ± 0.504 *vs.* 1.250 ± 0.250, and 39.50 ± 2.296 *vs.* 10.76 ± 1.123, respectively). Taken together, our data illustrated that KLF4 attenuated the migration and invasion of NSCLC cells *in vitro*.

### Overexpression of Krüppel-Like Factor 4 Attenuated TGF-B1-Induced Epithelial-Mesenchymal Transition and Inhibited the c-Jun-NH2-Terminal Kinase Signaling Pathway

**Figure 10:**
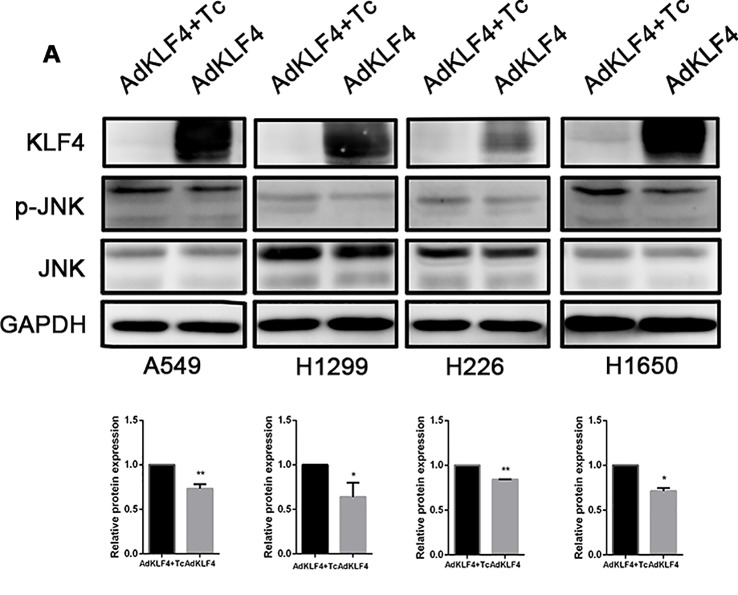
Overexpressed Krüppel-like factor 4 (KLF4) in non-small-cell lung cancer (NSCLC) cell lines inhibited the phosphorylation of c-Jun-NH2-terminal kinase (JNK). **(A)** Total protein was extracted from A549, H1299, H226, and H1650 cells. KLF4 was overexpressed in the AdKLF4 group. The protein levels of total and phosphorylated forms of JNK were compared in the indicated cells. GAPDH was used as a loading control. The experiment was repeated at least three times. The quantitation of p-JNK expression in each sample was normalized with that of JNK by determining the p-JNK/JNK ratio. Quantitative analysis demonstrating a significant decrease of p-JNK expression in the AdKLF4 group compared with AdKLF4+Tc group. The data were presented as mean ± SEM. *,** p < 0.05. AdKLF4: Lung cancer cells were transfected with adenovirus expressing KLF4. AdKLF4+Tc: Lung cancer cells were transfected with adenovirus expressing KLF4 and cultured with tetracycline.

Our previous published data demonstrated that overexpression of KLF4 attenuated TGF-β1-induced EMT in A549 (Lin et al., 2017). The JNK (c-Jun-NH2-terminal kinase) pathway plays important roles in inflammation, differentiation, apoptosis, insulin resistance, and cell migration. To examine the mechanism through which KLF4 attenuates the metastasis of lung cancer, the phosphorylation of JNK (p-JNK) in A549, H1299, H226, and H1650 cell lines was measured using Western blot, as shown in **Figure 10A**. Our results showed that KLF4 overexpression downregulated JNK phosphorylation in A549, H1299, H226, and H1650 cell lines. The statistical data revealed that the expression level of p-JNK of all cell lines was decreased in AdKLF4 group compared with the control group. These results indicated that the JNK/EMT signaling pathway might be involved in KLF4-suppressed migration and invasion of NSCLC cells.

## Discussion

Our results demonstrated that the expression of KLF4 was decreased in NSCLC; E-cadherin expression was decreased and vimentin expression was increased in NSCLC; KLF4 overexpression suppressed the invasion and metastasis of NSCLC by attenuating MET *in vivo*. KLF4 overexpression suppressed the migration and invasion of NSCLC cells *in vitro*. KLF4 overexpression suppressed the migration and invasion of NSCLC cells, possibly by inhibiting JNK/EMT signaling pathway.

KLF4, a notable transcription factor, has dual functions in transcriptional activation and suppression. Multiple researches showed that KLF4 was downregulated in colon cancer, lung cancer, esophageal cancer, bladder cancer, but upregulated in breast cancer. Our data showed that the expression of KLF4 was decreased in NSCLC tissues, which was consistent with the findings of other studies (Fadous-Khalife et al., 2016). Furthermore, as a novel discovery of this study that has not been previously reported, we found that the expression of KLF4 in metastatic lung cancer tissues in the trachea and main bronchus was downregulated. The underlying mechanism of the distinguished expression of KLF4 between tumor tissues and adjacent normal tissues is unclear, and some scholars found that hypermethylation of the promoter was associated with its transcriptional repression (Hu et al., 2009), while others thought *KLF4* was likely repressed by histone acetylation (Yu et al., 2016). Yu T et al. showed that KLF4 regulates adult lung tumor-initiating cells and HDAC3 has an inhibitory role on KLF4 promoter activity that might contribute to the downregulation of KLF4 in lung cancer. This issue needs further exploration and discussion in future work.

KLF4 plays an important role in tumor initiating, growth, and metastasis. Ghaleb AM et al. found that KLF4 suppressed the development of colonic neoplasia (Ghaleb et al., 2016). Chuan Tian et al. revealed that KLF4 overexpression inhibits the growth and metastasis of hepatocellular carcinoma cells (Tian et al., 2017). Tango H et al. demonstrated that KLF4 was downregulated in anaplastic meningioma compared with low-grade meningiomas, and KLF4 could reduce the invasive ability of anaplastic meningioma stem-like cells (Tang et al., 2017). Yang Y et al. found that KLF4 increased with advanced cancer stage and promoted invasion of human esophageal squamous cell cancer cells (Yang and Katz, 2016). A meta-analysis of 2,988 patients showed that low KLF4 expression was related to worse overall survival and disease-free survival in solid tumor (Yu et al., 2018). KLF4 has been shown to be associated with the aggressiveness of many types of cancer and was also reported to be a tumor suppressor gene in lung cancer. Yu et al. found that the KLF4 deletion facilitated tumor formation in mouse lungs with K-ras activation (Yu et al., 2016). Hu et al. revealed that KLF4 suppressed the growth of human lung cancer cell lines by regulating the cell cycle and cell proliferation (Hu et al., 2009). However, the role of KLF4 in lung cancer metastasis remains unclear. Zhou et al. discovered that KLF4 inhibited lung cancer cell invasion by suppressing SPARC gene expression (Zhou et al., 2010). However, Shi et al. discovered that a deficiency of KLF4 in mouse bone marrow decreased the incidence of lung metastasis (Shi et al., 2014).We found that the E-cadherin expression was decreased, while the vimentin expression was increased in NSCLC tissues and metastatic lung cancer tissues in the trachea and main bronchus. We assume that EMT (MET) was involved in the KLF4 suppression of the metastasis process. We found that the overexpression of KLF4 in mouse lungs using an intratracheal injection of AAV5-KLF4 could decrease the incidence of lung metastasis. LLC cell line is widely used as a model for metastasis. In this study, we used IVIS in conjunction with D-luciferin to track the invasion of LLC cells, which is a direct and sensitive method to observe metastasis (Mezzanotte et al., 2017). The bioluminescence from luciferase-gene transfected cancer cells is often used in tumor-bearing mouse models. We used bioluminescence to observe metastasis in mouse lungs. We also performed triple-labeled immunofluorescence staining in mouse lung tissue sections to observe the cells undergoing MET. An EMT can promote cancer cell migratory and invasive abilities; and its reverse process, a MET that seems to support metastatic outgrowth in distant organs (Thiery et al., 2009). Our co-staining results revealed that KLF4 overexpression could reduce the number of cells undergoing MET. The ectopic overexpression of KLF4 in mouse lungs contributed to the decreased incidence of lung metastasis by attenuating MET. This phenomenon was likely due to the formation of a local microenvironment with KLF4 overexpression in the lung but needs further investigation. *In vitro* studies, we used four different cell lines to observe how KLF4 overexpression influences the migratory and invasive ability. A549 is epithelial-like lung cancer cells from lung carcinomatous tissues. NCI-H1299 was established from a lymph node metastasis of the lung from a patient who had NSCLC and received prior radiation therapy. NCI-H226 was derived from the pleural effusion of patient who had squamous cell carcinoma and mesothelioma, and metastasized to pleural cavity. NCI-H1650 was derived from pleural effusion of patient who had stage 3B adenocarcinoma, and bronchoalveolar carcinoma, and metastasized to pleural cavity. Our results revealed that KLF4 overexpression could inhibit migration and invasion of both primary and metastatic carcinomatous cells.

Recently, the role of JNK has been acknowledged in physiological and pathological states. JNK belongs to the mitogen-activated protein kinase (MAPK) family of proteins, and includes three isoforms, JNK1, JNK2 and JNK3. The JNK MAPK pathway is primarily activated by cytokines and an exposure to environmental stress. The activation of this pathway requires the phosphorylation of threonine and tyrosine residues (Kyriakis et al., 1991). The JNK MAPK pathway is involved in many cellular processes, such as cell growth, differentiation, migration, apoptosis, and inflammatory and immune responses. It has also been implicated in many pathological conditions, including cancer, stroke, heart disease, and inflammatory disease (Coffey, 2014; Yuan et al., 2017; Blum et al., 2019; Cosolo et al., 2019). Studies show that JNK plays a vital role in the whole metastatic process. JNK affects the local invasion of tumor cells and the survival, homing and extravasation of circulating tumor cells (Ebelt et al., 2013). Previous studies have demonstrated that activated JNK promotes the invasion and metastasis of tumors by promoting the development of EMT (Zhao et al., 2018; Wang et al., 2018; Dong et al., 2018). Choi Y et al. reported that JNK inhibition suppressed migratory capacity through reversing EMT in gastric cancer (Choi et al., 2016). JNK has also been reported to promote EMT in renal cell carcinoma (An et al., 2015). TGF-β1 triggers EMT in lung cancer *via* the JNK pathway (Khan et al., 2018). Our previous published data demonstrated that overexpression of KLF4 attenuated TGF-β1-induced EMT in A549 (Lin et al., 2017), which were consistent with other publications (Liu et al., 2016). Our data showed that the overexpression of KLF4 can cause a downregulation of p-JNK, which indicate that the overexpression of KLF4 contributed to the decreased ability of migration and invasion through JNK/EMT signaling pathway. The limitations of our work are that the mechanistic study is weak. Therefore, in the future, our main goal is to explore the possible interaction and signaling pathway between KLF4, EMT, and p-JNK and develop an understanding of how they function in NSCLC metastasis.

In conclusion, the overexpression of KLF4 inhibited the metastasis of NSCLC *in vivo* by attenuating MET. KLF4 overexpression suppressed the migration and invasion *in vitro* by attenuating JNK/EMT signaling pathway. KLF4 is a potential novel tumor suppressor in NSCLC and may be a promising therapeutic target.

## Data Availability Statement

All datasets generated for this study are included in the article.

## Ethics Statement

The studies involving human participants were reviewed and approved by the Clinical Research Ethics Committee of the Peking University First Hospital. The patients/participants provided their written informed consent to participate in this study. The animal study was reviewed and approved by the Animal Research Committee of the Peking University First Hospital.

## Author Contributions

YW performed the experiments, analyzed the data, prepared the figures, wrote the manuscript, and completed the review process. LLi contributed to the conception and design of the experiments and manuscript preparation. XW, YL, ZL, WH, GL, HL, and JL helped with the collection of human non-small cell lung cancer samples. WY and LLv helped with the collection of metastatic tumor tissues located in the trachea and main bronchus. JZ and TL performed the pathological diagonosis. BZ and NW helped with the analysis with constructive discussions. XL contributed to the conception and design of the study.

## Funding

This work was supported by the National Natural Science Foundation of China [grant number 81670043].

## Conflict of Interest

The authors declare that the research was conducted in the absence of any commercial or financial relationships that could be construed as a potential conflict of interest.
